# Protein–Polysaccharide Bilayer Films: Influence of Protein and Cross-Linker on Interfacial and Functional Properties

**DOI:** 10.1021/acs.biomac.5c02422

**Published:** 2026-02-05

**Authors:** Giuliana T. Franco, Luana Figueiredo, Caio G. Otoni, Luiz H. C. Mattoso

**Affiliations:** † Nanotechnology National Laboratory for Agriculture (LNNA), 67838 Embrapa Instrumentation − Rua XV de Novembro, 1452, São Carlos, SP 13561-206, Brazil; ‡ Graduate Program in Chemistry (PPGQ), Federal University of São Carlos (UFSCar) − Rod. Washington Luís, km 235, São Carlos, SP 13565-905, Brazil; § São Carlos Institute of Chemistry (IQSC), University of São Paulo (USP), Av. Trabalhador São Carlense, Parque Arnold Schimidt, São Carlos, SP 13566-590, Brazil; ∥ Graduate Program in Materials Science and Engineering (PPGCEM), Federal University of São Carlos − Rod. Washington Luís, km 235, São Carlos, SP 13565-905, Brazil; ⊥ Institute of Chemistry, University of Campinas (Unicamp), Rua Monteiro Lobato, 270, Campinas, SP 13083-862, Brazil

## Abstract

Self-supporting bilayer films were produced by combining a protein (casein or gelatin) and a polysaccharide (carboxymethylcellulose, CMC). Effective interfacial interactions, achieved either physically (primarily via electrostatic interactions) or chemically (with citric acid and 1,2,3,4-butanetetracarboxylic acid (BTCA) as cross-linkers), granted improved mechanical and functional performance. Gelatin layers exhibited strong adhesion across different pH values (3, 4.5, and 8), indicating a minimal role of electrostatic forces in interlayer interactions. In contrast, casein required the incorporation of tannic acid (TA) into the CMC layer as a compatibilizing agent to achieve effective adhesion. Polysaccharide cross-linkers were evaluated in casein-CMC bilayers, where citric acid reduced the water absorption, while BTCA improved water vapor barrier properties but decreased mechanical resistance. Understanding interfacial interactions enables the design of biobased materials with tailored properties, boosting competitiveness and functionality within the circular bioeconomy.

## Graphical abstract

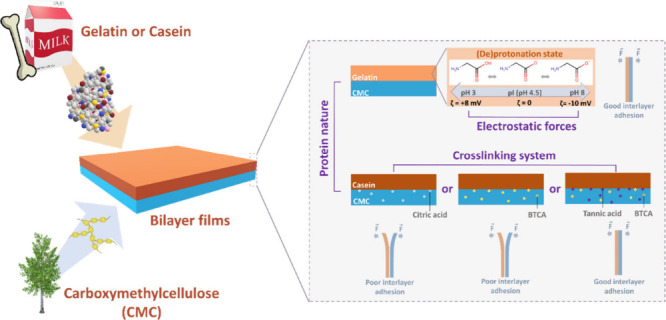

## 1 Introduction

Films derived from natural polymers like proteins and polysaccharides offer a practical alternative to fossil-based and nonbiodegradable polymers.^1^ Besides their abundance and biodegradability, these biopolymer-based films can be processed,^2^ chemically modified,^3^ and combined with other components to enhance and tailor their properties.^4^ However, challenges such as high costs and limited scalability hinder their market adoption and large-scale production.^5^ Approaches like developing multistructured materials, blending polymers, adding additives, and scaling up can improve cost-efficiency, enhance processability, and optimize properties.^1,6^ Bilayer films of proteins and polysaccharides combine the beneficial features of both components, improving performance and enabling tailored materials.^7,8^ Pasquier et al.^9^ developed a multilayer film composed of cellulose nanofibers (CNF), wax particles (WP), and lignin particles integrated with chitin nanofibers (LPChNF), which demonstrated barrier properties against UV light, water vapor, and oxygen. Each layer plays a complementary role, increasing the film’s overall protective effectiveness.

These biopolymers primarily interact through electrostatic forces and hydrogen bonding.^10^ Gelatin is a protein derived from the partial hydrolysis of collagen, which is obtained from the skin, bones, and connective tissue of animals.^11^ The secondary structure can be partially restored when water is added because of the remaining collagen fibers, which trigger gelation.^12^ Casein is the main milk protein that organizes into micelles due to its amphiphilic nature, stemming from hydrophilic and hydrophobic amino acids. Classified as an intrinsically disordered protein (IDP), casein consists mostly of its primary structure and remains stable without denaturing when heated.^13,14^ Carboxymethylcellulose (CMC) is an anionic, film-forming polysaccharide derived from cellulose through hydroxyl substitution with carboxymethyl groups.^15^ Gelatin and casein form stable complexes with CMC, enabling their use in nanocapsules to deliver active compounds such as eugenol,^16^ urea,^17^ and anthocyanins.^18,19^ Gelatin has been shown to alter its secondary structure into a more flexible form through interaction with CMC and chitosan.^16^ Additionally, CMC is often used to stabilize casein in acidic conditions where it would otherwise be unstable by changing the charge density of the system.^20,21^ 1,2,3,4-Butanetetracarboxylic acid (BTCA) and citric acid serve as polysaccharide cross-linkers because they can form ester bonds between hydroxyl groups. BTCA is a more effective cross-linker because of its four carboxyl groups, whereas citric acid, with three carboxyl groups, creates a less rigid network but offers greater biocompatibility and cost-effectiveness.^22,23^

The combination of CMC with casein or gelatin in a bistructured film represents a strategy to integrate the mechanical strength provided by CMC with the modulation of moisture transmission and the more hydrophobic and cohesive characteristics of the protein layer, resulting in a more balanced and functional material for applications, such as food packaging.^9,24^ When polysaccharides and proteins are combined in solution, without forming insoluble coacervates or phase separation,^8,25^ a continuous two-dimensional interface is established in the bilayer films. Effective interfacial interactions are essential for ensuring interlayer adhesion and determining the desired properties of the bistructured material.^26^ In some cases, a compatibilizing agent is necessary to increase the affinity between these components. Tannic acid (TA) is a polyphenol rich in hydroxyl groups with high efficiency in binding proteins and polysaccharides.^10,27^ Shan et al.^28^ incorporated TA and silver nanoparticles into the protein layers of a gelatin hydrogel/ethyl cellulose bilayer film. The polyphenol facilitated the formation of a denser hydrogel network, enhancing the humidity-responsive properties of the films and improving their oxygen barrier performance.

Here, a self-supporting bilayer film was produced by combining a protein (casein or gelatin) layer with a polysaccharide (CMC) layer using continuous casting, an upscaling method. This bistructured configuration was employed as a strategy to integrate polysaccharide and protein matrices through a single, continuous interface, providing a simplified yet representative model system that enables a systematic investigation of how interfacial adhesion, compatibility, and cross-linking affect material properties. First, the affinity between CMC and the protein was explored. The effect of electrostatic forces was studied in gelatin-CMC (GEL-CMC) bilayer systems, in which films were produced from gelatin solutions at pH 3 (positively charged), pH 4.5 (neutral), and pH 8 (negatively charged). The composition of the CMC layer was evaluated by changing the polysaccharide cross-linker (citric acid or BTCA) in casein-CMC (CA-CMC) films. Due to poor adhesion between casein and CMC layers, TA was tested as a compatibilizer. Moreover, the use of a scalable processing technique provides self-supporting bistructured films with controlled thickness and suitable optical transparency, comparable to commercially available packaging films, demonstrating their relevance and the potential for sustainable applications.

## 2 Materials and Methods

### 2.1 Materials

Carboxymethylcellulose sodium salt, CMC (USP; Synth, Brazil; degree of substitution: 0.95 ± 0.09); gelatin, GEL (80% pure; Êxodo Científica, Brazil); casein sodium salt, CA (80% pure; Exôdo Científica, Brazil); glycerol (99.5% pure; Dinâmica, Brazil); tannic acid, TA (ACS; Sigma-Aldrich, China); 1,2,3,4-butanetretacarboxylic acid, BTCA (99% pure; Sigma-Aldrich, India); citric acid (99.5% pure; Vetec, Germany); sodium hypophosphite, SHP (99%; Synth, Brazil); and copper sulfate hydrated (99% pure; Synth, Brazil) were used as received. Ultrapure water, deionized (ρ = 18.2 MΩ cm) in a Milli-Q system (Barnstead Nanopure Diamond, USA), was used in all experiments

### 2.2 Film-Forming Solutions

The CMC layers were produced by solubilizing the polymers in water to obtain a 2 wt % solution on a wet basis (w.b.). The cross-linker – citric acid or BTCA – and the catalyst, sodium hypophosphite, were added at a 2:1 weight ratio, both predissolved in 3 mL of water to obtain a concentration of 15 wt % on a dry basis (d.b.), and stirred for 20 min. For the films containing TA, 5 wt % (d.b.) of the polyphenol was added, predissolved in 3 mL of water, and stirred for an additional 20 min. Finally, 15 wt % (d.b.) glycerol was added, and the mixture was stirred for an additional 30 min. For protein layers, casein or gelatin was solubilized in water at 20 wt % (w.b.). Glycerol at 30 wt % (d.b.) was incorporated, and the solution was stirred for another 30 min. Casein was solubilized at pH > 5, and the final pH was adjusted to 8. Gelatin solutions were adjusted to pH 3.0, 4.5, or 8.0 using 1 M aqueous HCl or NaOH solutions. Except for the gelatin solution, all solutions were centrifuged at 10,000 rpm for 10 min at 25 °C to remove air bubbles.

### 2.3 Continuous Casting of the Bilayer Films

Self-supporting protein-CMC films were produced through two continuous lamination steps on a KTF-S coater machine (Werner Mathis AG, Switzerland). Initially, the CMC solution was poured onto a polyester substrate (Mylar, DuPont, Brazil), which moved at 0.07 m.min^–1^ toward a doctor blade device set at a 1.25 mm gap from the substrate (wet thickness). The film then passed through two convective 1-m-long ovens at 95 °C for drying. Next, the protein layer—either casein or gelatin—was laminated onto the dried CMC layer, which moved at 0.1 m.min^–1^ toward the doctor blade set at 0.8 mm. The film then went through two convective ovens at 70 °C. The CMC layers combined with casein or gelatin were designated as CA-CMC and GEL-CMC, respectively. The films GEL_pH3_-CMC_C_, GEL_pH4.5_-CMC_C_, and GEL_pH8_-CMC_C_ refer to systems produced using gelatin solutions adjusted to pH 3.0, 4.5, and 8.0, respectively. For the films CA-CMC_C_, CA-CMC_B_, and CA-CMC_BT_, the letters C, B, or BT indicate the addition of citric acid, BTCA, or BTCA with TA, respectively, into the CMC layer.

### 2.4 Zeta Potential Measurements

The apparent zeta potential was measured using a ZEN 3600 instrument (Malvern Instruments, USA) with aqueous solutions at 0.1 wt % (w.b.) over a pH range of 2 to 11, adjusted with aqueous 1 M HCl or NaOH solutions.

### 2.5 Rheological Analysis

The shear viscosity as a function of shear rate (0 to 1000 s^–1^) was evaluated at 25 °C using a rheometer MCR 301 (Anton Paar, Austria) equipped with a coaxial cylinder cell (DG26.7). Gelatin and casein solutions were analyzed at 20 wt % (w.b.), while CMC solutions were studied at 3 wt % (w.b.). The flow index (*n*) was determined by eq 1.

1 $$\tau =\tau_{0}+k\gamma^{n}$$

where τ is the shear stress [Pa], τ_0_ is the yield stress [Pa], *k* is the consistency index, γ is the shear rate [s^–1^], and *n* is the flow index.

### 2.6 Quartz Crystal Microbalance with Energy Dissipation (QCM-D) Measurements

In QCM-D experiments, 30 mL of aqueous solution of CMC at 0.05 wt % (w.b.) or proteins at 0.1 wt % (w.b.) for proteins were injected at a flow rate of 0.1 μL·min^–1^, alternated with water (with pH adjusted to match the protein solutions), as follows: water (15 min), protein (30 min), water (15 min), CMC (30 min), and water (15 min). The pH of the casein solution was adjusted to 8.0, while those of the gelatin solutions were adjusted to 3.0, 4.5, and 8.0, as described above. The QCM-D experiments were performed using gold sensors with a fundamental frequency of 5 MHz, monitoring 13 harmonics. Previously, the sensors had been cleaned in a ProCleaner UV/O_3_ chamber (Bioforce Nanoscale, USA) for 15 min.

### 2.7 Qualitative Delamination Tests

Bilayer films were cut into 2 cm × 6 cm specimens, and a high-adhesion tape (Scotch 9400-3M double-sided tape) was glued to each face. The tape was manually pulled off in opposite directions to observe delamination.

### 2.8 Field Emission Scanning Electron Microscopy (FE-SEM)

Cross-sectional and surface images of the films were registered using a JMS 6510 scanning electron microscope (JEOL, Japan). For cross-sectional images, the bilayers were cryo-fractured under liquid nitrogen and fixed at 90° onto aluminum stubs. Both surfaces and fractures were coated with a thin platinum layer before being imaged at 2–5 kV.

### 2.9 Equilibrium Moisture Content (EMC)

The EMC of the bilayer films was evaluated by keeping samples (5 cm × 5 cm) in an airtight desiccator containing a saturated copper sulfate solution (RH = 90%, 25 °C). The samples were weighed before and after 24 h.^29^ The EMC was calculated by eq 2.

2 $$\mathrm{EMC}\left(\%\right)=\frac{m_{f}-m_{i}}{m_{i}}\times 100\%$$

where EMC is the equilibrium moisture content [%], *m*_i_ is the initial mass [g], and *m*_f_ is the final mass [g].

### 2.10 Water Vapor Permeability (WVP)

The WVP of the bilayers was determined through a gravimetric method based on an adaptation of ASTM E96–80.^30^ Films (2 cm × 2 cm) were mounted onto a sealed PTFE cup (2.4 cm internal diameter) containing 2 mL of water. These cups were kept in a controlled environment chamber (Ethik, model 420–2TS 295L, Brazil) set at 25 °C and 50% RH. The cups were weighed hourly for 6 h, then once after 24 h. The WVP was calculated by eq 3:

3 $$\mathrm{WVP}=\frac{\mathrm{WVTR}\times L}{\Delta P}$$

where WVP is water vapor permeability [g·m·h^–1^·Pa^–1^], WVTR is water vapor transmission rate [g·h^–1^], *L* is the average thickness [mm], and Δ*P* is the pressure difference [kPa].

### 2.11 Water Contact Angle

The surface wettability properties of each bilayer film face were investigated by contact angle measurements using the sessile drop method on a Theta Lite tensiometer (Biolin Scientific, Sweden). Water (300 μL) was dropped onto the film surface, and images were recorded for 60 s. The average contact angle was calculated as the arithmetic mean of the right- and left-droplet angles. Four replicates were performed for each sample.

### 2.12 Mechanical Properties

The mechanical properties were evaluated through a uniaxial tensile test in accordance with ASTM D638-14.^31^ Film specimens were conditioned at 25 °C and 50% RH for 24 h, and the tests were carried out on a DL-300 universal machine (EMIC, Brazil) equipped with an 18 N load cell at a rate of 0.1%·min^–1^. Film thickness was measured using a digital micrometer (Mitutoyo Manufacturing, IP 65, Japan) with an accuracy of 0.001 mm at five points, and the values were used to calculate the mechanical and water barrier properties.

### 2.13 Thermogravimetric Analysis (TGA)

The thermogravimetric curves were obtained using a Q500 instrument (TA Instruments, USA). About 10 mg of each sample was weighed in a platinum pan and heated from 25 to 500 °C at 10 °C·min^–1^ under a synthetic air atmosphere flowing at 60 mL·min^–1^.

### 2.14 Statistical Analysis

The data were analyzed using analysis of variance (ANOVA) and Tukey’s test at a 5% significance level (*p* = 0.05) with Minitab software, version 18 (LLC, USA).

## 3 Results and Discussion

### 3.1 Study of Intermolecular Interactions in Aqueous Solution

The chemical structures of the biopolymers and cross-linkers studied are shown in Figure 1, while the zeta potential as a function of pH and rheological properties for protein and CMC solutions are shown in Figure 2. CMC presented a negative apparent zeta (ζ) potential above its p*K*_a_ (Figure 2a) due to its deprotonated carboxyl groups. The film-forming CMC solution naturally had an acidic pH (pH ∼ 3), with a slightly negative ζ potential (ζ = −10 mV). As gelatin is a hydrolyzed protein that can restructure into a helix when hydrated, surface charges can affect electrostatic intermolecular forces and interfacial interactions with CMC.^25^ Thus, gelatin solutions were adjusted to pH (below the isoelectric point, pI; ζ = +9 mV), 4.5 (pH = pI; ζ = 0), and 8 (pH > pI; ζ = −11 mV). The casein solution had the pH adjusted to 8 (ζ = −14 mV), because it coagulates below the isoelectric point (pH ∼ 5) and exhibits minimal variation in ζ potential above pH 8. Given the similar ζ magnitude of casein (pH 8) and CMC (pH ∼ 3) solutions used for film productions, repulsive forces may hinder their interaction.^32^

**Figure 1:**
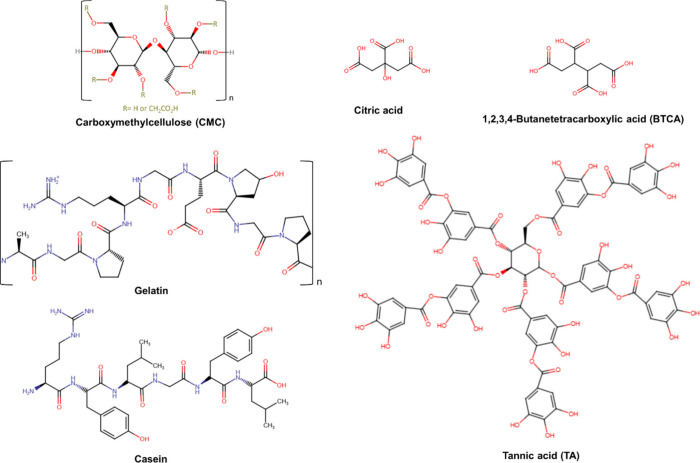
Representation of the idealized chemical structures of the biopolymers carboxymethylcellulose (CMC), gelatin, and casein, and the cross-linkers citric acid, 1,2,3,4-butanetetracarboxylic acid (BTCA), and tannic acid (TA).

**Figure 2:**
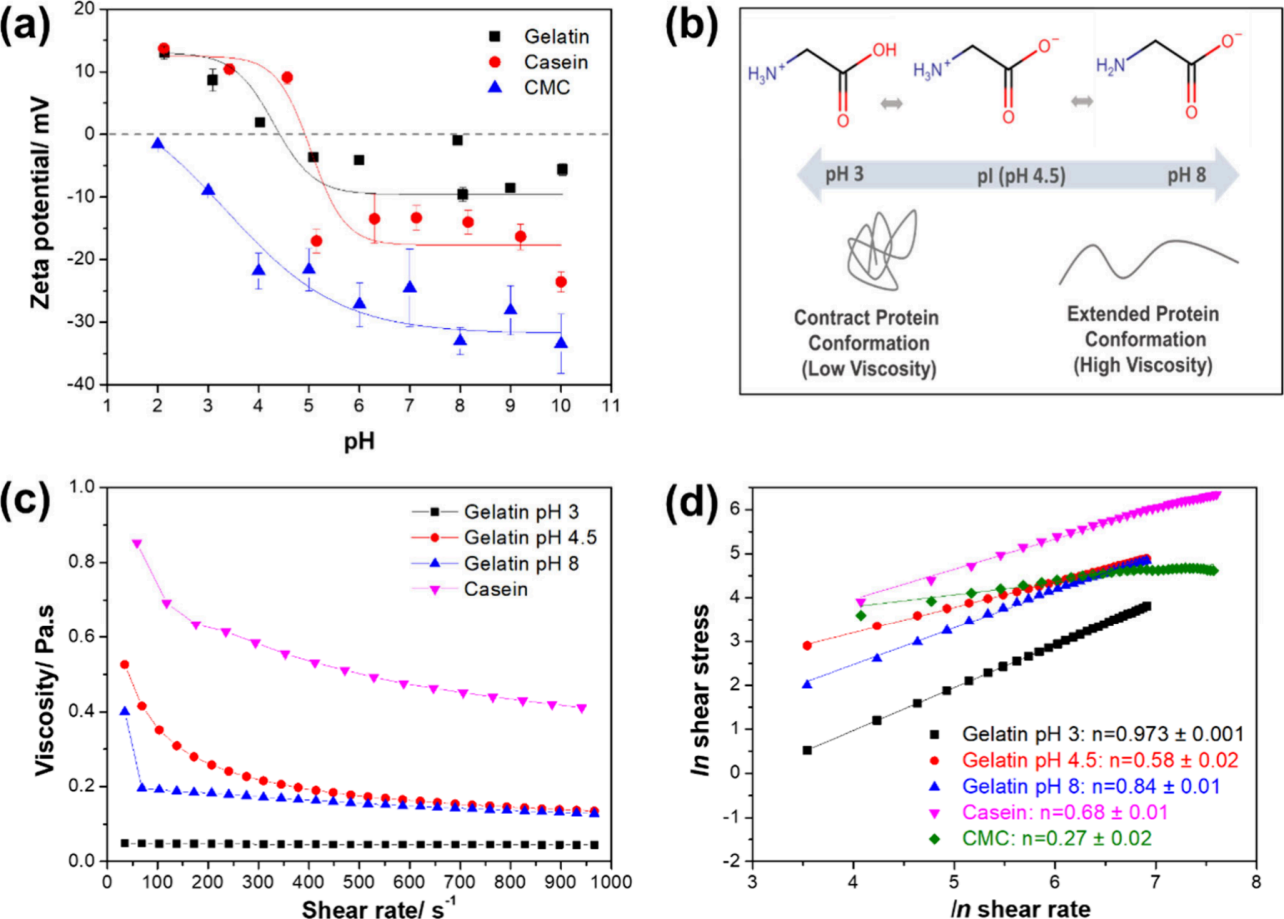
(a) Apparent zeta potential as a function of pH, (b) schematic of protein chain conformation in relation to pH, (c) viscosity profiles as a function of shear rate, and (d) logarithmic curves of shear stress as a function of shear rate (*n* = flow behavior index) for aqueous carboxymethylcellulose (CMC), casein, and gelatin solutions at pH 3, 4.5, and 8.

The flow behavior of polymer solutions is an important parameter, as it directly impacts the spreadability, thickness, and uniformity of coatings during lamination.^33^ As shown in Figure 2b,c, the viscosity of film-forming solutions decreases with increasing shear rates up to approximately 400 s^–1^ (Figure 2b), after which it levels off, except for the gelatin solution at pH 3, which maintains a constant viscosity within the analyzed range. This decrease in viscosity reflects the progressive disentanglement and alignment of polymer chains in the flow direction, a process driven by repulsive forces and leading to the shear-thinning effect.^34^ The logarithmic curve (Figure 2c) illustrates the rheological behavior of the solutions, with gelatin solutions at pH 3 and 8 showing behavior close to Newtonian (*n* = 1), meaning viscosity was rather independent of shear rate. Surface charges influenced chain cohesion and molecular conformation in gelatin solutions at pH 3 and 8 because of electrostatic repulsion, resulting in a more open, less entangled structure with a larger hydrodynamic volume. Consequently, less shear force is needed to align the polymer chains.^35,36^ Gelatin at pH 4.5 and casein solutions exhibit pseudoplastic flow behavior (*n* < 1), indicating greater entanglement.^33,37^ Although casein carries a net negative charge at the pH used, its phosphoprotein nature leads to micelle formation stabilized mainly by hydrophobic interactions, which hinders an open conformation.^38^ The CMC solution also shows pseudoplastic behavior (*n* < 1), similar to gelatin at pH 4.5, despite being much less concentrated.

QCM-D experiments were performed to examine the affinity between the CMC and the proteins, as shown in Figure 3. Shifts in resonance frequency indicate mass adsorption or desorption, while changes in dissipation relate to the viscoelastic properties of the sensor-coating film.^39^ Introducing the initial protein solutions decreased frequency (Figure 3a), and their magnitudes varied depending on the type of protein and the pH of the gelatin solution, reflecting different capacities for adsorption on the sensor surface.^40^

**Figure 3:**
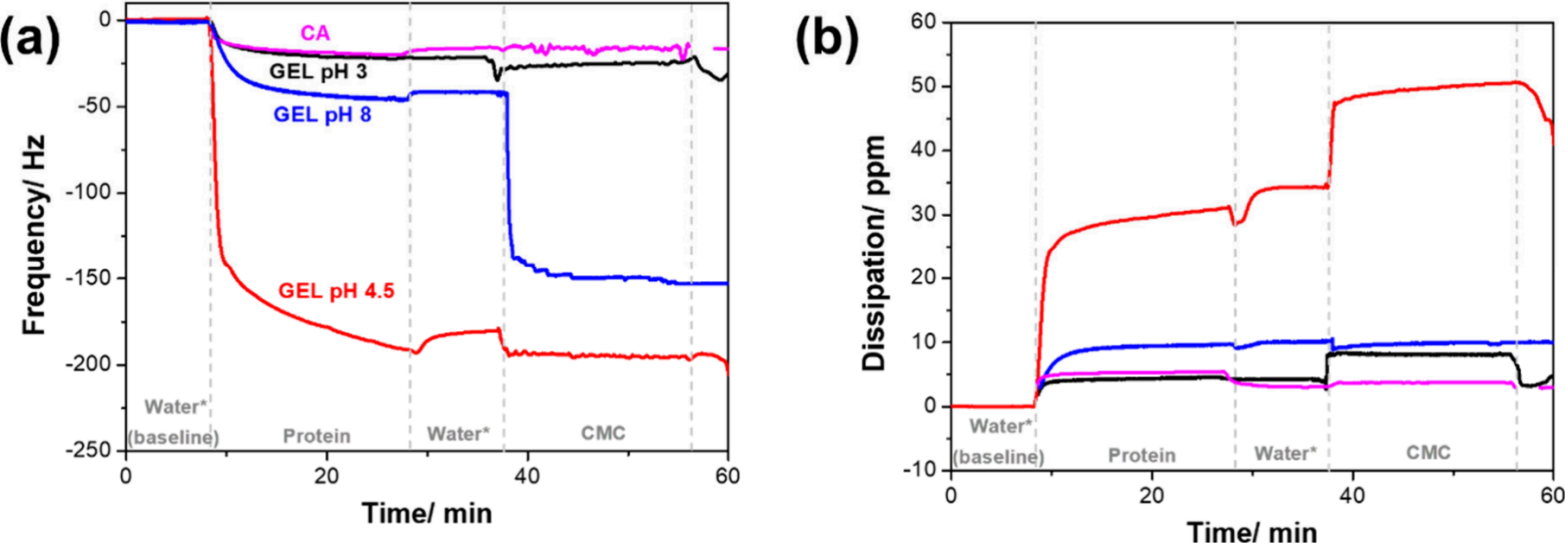
Normalized curves of (a) third overtone frequency and (b) energy dissipation as a function of time from QCM-D analysis. The data illustrate four sequential injection steps: water (baseline), protein solution, water rinse, and carboxymethylcellulose (CMC) solution. *The water pH was adjusted to match the protein solution, with pH values of 3, 4.5, or 8 for gelatin (GEL) and pH 8 for casein (CA).

The frequency and dissipation shift of interest occur between the protein and CMC injections (Δ*f*_CMC‑protein_), which relate to the affinity between these components (Table S1). CMC showed the highest interaction with gelatin at pH 8 (negatively charged), as indicated by the largest frequency shift (Δ*f*_CMC‑protein_ = −107 Hz). The frequency shifts for gelatin at pH 3 (positively charged) (Δ*f*_CMC‑protein_ = −1 Hz) and pH 4.5 (neutral charged) (Δ*f*_CMC‑protein_ = −4 Hz) were smaller, indicating low adsorption of CMC and weak affinity to the protein layer. A similar low interaction was observed between CMC and casein (Δ*f*_CMC‑protein_ = −4 Hz), in which the protein is also negatively charged but behaved differently compared to gelatin at pH 8. Opposite charges did not lead to stronger interactions, which would be expected in aqueous media, as observed for the gelatin at pH 3/CMC system. This suggests that electrostatic attraction is not the main contributor to the affinity between CMC and casein/gelatin, and that interactions are likely influenced by structural composition and conformation of the proteins. At higher pH levels, proteins undergo intramolecular repulsions that cause them to unfold, exposing internal hydrophobic regions and nonpolar groups, which increases the number of active sites available to interact with CMC or surfaces.^41,42^ These findings align with those of Li et al.,^43^ who found that whey protein deposition on a 1-hexadecanethiol-modified gold surface depends on the ionic strength of the surrounding environment.

The dissipation shifts between CMC and proteins (Δ*D*_CMC‑protein_; Figure 3b) revealed that CMC deposition on gelatin at pH 3 (Δ*D*_CMC‑protein_ = 4 ppm) or pH 8 (Δ*D*_CMC‑protein_ = 0), as well as on casein (Δ*D*_CMC‑protein_ = −2 ppm), had minimal effects on the viscoelastic properties of the systems. However, deposition on gelatin at pH 4.5 (Δ*D*_CMC‑protein_ = 20 ppm) caused significant energy dissipation, indicating the formation of a soft layer.^43^ The ratio of dissipation to frequency shifts (Δ*D*/(−Δ*f*)) allows for comparison of energy dissipation per unit mass (Table S1), where lower values of Δ*D*/(−Δ*f*) suggest more rigid adsorbed layers.^44^ Gelatin at pH 4.5/CMC showed the highest Δ*D*/(−Δ*f*) value (5 ppm·Hz^–1^), reflecting the most viscoelastic system, followed by gelatin at pH 3/CMC (3 ppm·Hz^–1^). Conversely, gelatin at pH 8/CMC (0.003 ppm·Hz^–1^) and CA/CMC (0.4 ppm·Hz^–1^) formed more rigid layers. The viscoelastic properties of polymers are mainly affected by their structural organization.^43^ The charge of gelatin modulates chain unfolding through repulsive forces,^42^ leading to different protein conformations and interactions with other molecules. Additionally, the micellar structure of casein can impact the properties of the formed layer and its interaction with the polysaccharide.^21^ Although CMC exhibited varying affinities for gelatin at different pH levels, the bilayer films created by continuous casting showed similar interlayer adhesion. In contrast, the bistructured films composed of CMC and casein, without any compatibilizer, displayed low adhesion.

### 3.2 Interfacial Interactions in the Bilayer Films

The bilayer films produced are continuous, self-supporting, and easy to handle, as shown in Figure 4a,b. A qualitative delamination test was conducted to evaluate the adhesion between the layers (Figure 4c). The gelatin and CMC layers were so tightly adhered that they could not be manually separated (Figure 4d–f). However, CA-CMC_C_ and CA-CMC_B_ films were easily delaminated (Figures 4g,h), indicating poor interlayer adhesion. In contrast, the layers of CA-CMC_BT_ could not be separated (Figure 4i).

**Figure 4:**
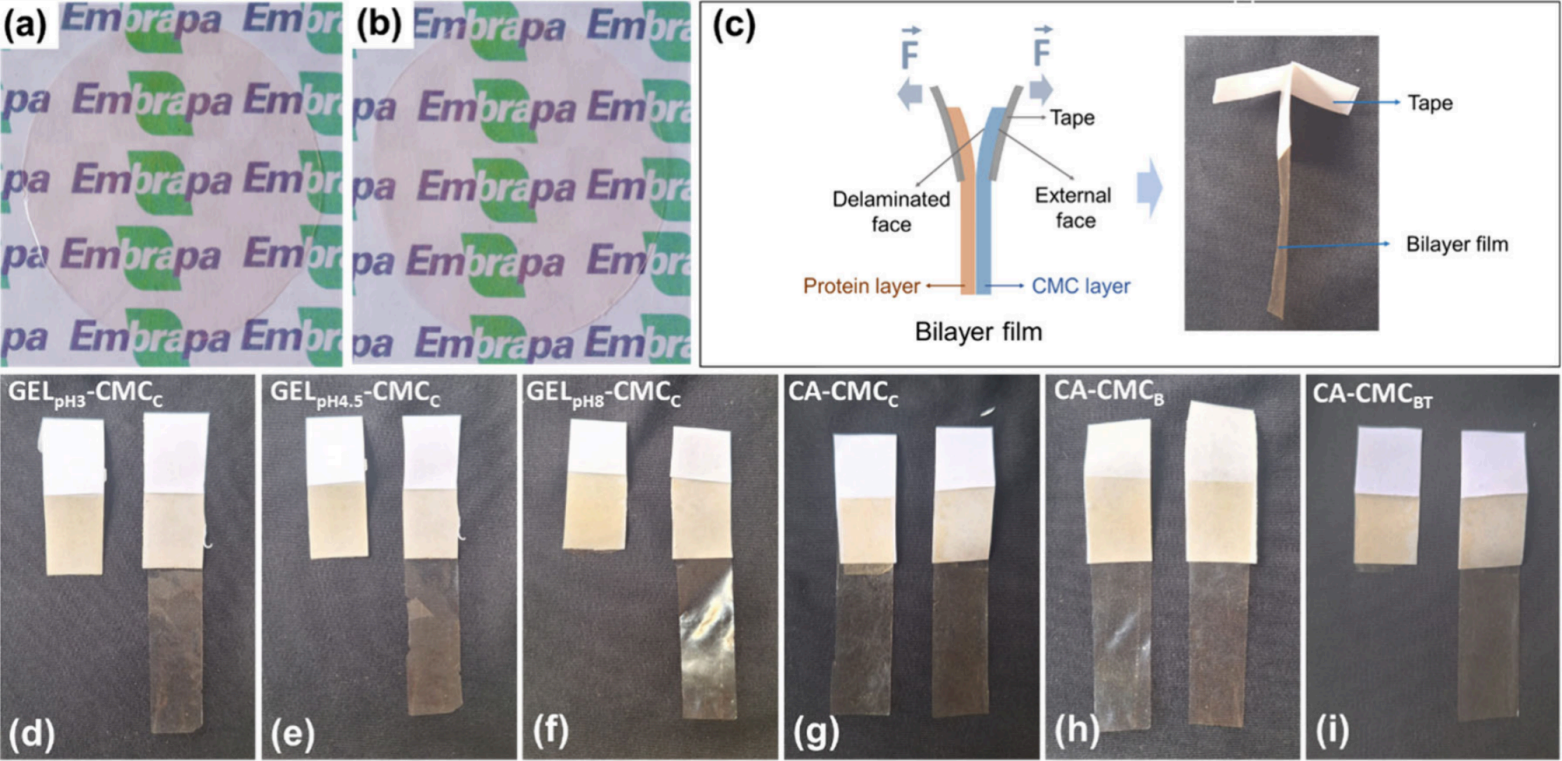
Digital images of (a) GEL_pH3_-CMC_C_ and (b) CA-CMC_C_ bilayer films. (c) Schematic representation of the qualitative delamination test, followed by the corresponding results for (d) GEL_pH3_-CMC_C_, (e) GEL_pH4.5_-CMC_C_, (f) GEL_pH8_-CMC_C_, (g) CA-CMC_C_, (h) CA-CMC_B_, and (i) CA-CMC_BT_ bilayer films.

TA (Figure 1) served in the CMC layer as anchoring points for the casein layer, improving interaction between the polymeric matrices.^45,46^ According to the literature, TA is oxidized in quinone intermediates, which bind to proteins, particularly lysine, tyrosine, and cysteine residues, forming covalent bonds via Michael addition or Schiff base reactions (Figure S1). While the ideal conditions for cross-linking with various proteins are not always clear, oxygen is generally considered essential for these reactions.^23^ Its presence may promote interfacial cross-linking with proteins at the film interface, as evidenced by the significant interlayer adhesion observed. Our previous work^46^ showed that CA–CMC films containing only BTCA in the CMC layer required a delamination force of approximately 0.06 ± 0.02 N, whereas films incorporating both BTCA and TA exhibited a delamination force of 0.16 ± 0.04 N, representing a significant increase. Given the high availability of hydroxyl groups in TA (Figure 1), this polyphenol can interact with CMC chains primarily via secondary forces, such as hydrogen bonding, hydrophobic interactions, and electrostatic interactions.^46^

Figure 5 shows the cross-sectional SEM images of the bilayer films. The bilayer films exhibited an average thickness of 0.05 ± 0.004 mm for the GEL-CMC systems and 0.05 ± 0.001 mm for CA-CMC systems. The individual gelatin, casein, and CMC layers showed average thicknesses of 0.03 ± 0.001 mm, 0.02 ± 0.002 mm, and 0.02 ± 0.005 mm, respectively. These thickness values are consistent with the morphological features observed in the SEM cross-sectional images. GEL-CMC films (Figure 5a–c) exhibit a uniform and continuous appearance across the cross-section, making it difficult to visually identify the interfacial region between the layers. In contrast, for CA-CMC systems (Figure 5d–f), each layer can be distinguished by its distinct appearance. The micrographs support the delamination test results, indicating that the CMC layers interact more with gelatin than with casein, with a well-defined interfacial line. No cracks, bubbles, or holes were observed in the region between the layers, which helps maintain effective interfacial contact and material integrity.^47^

**Figure 5:**
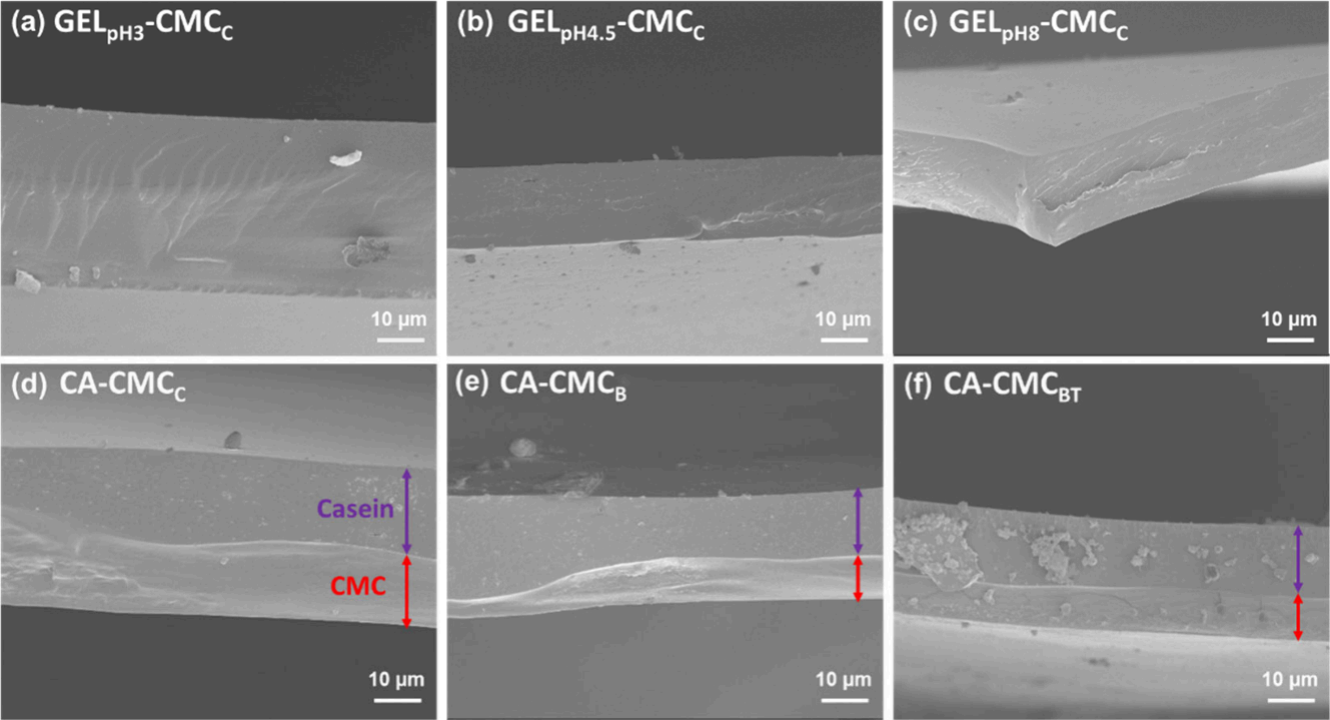
Cross-sectional SEM images of (a) GEL_pH3_-CMC_C_, (b) GEL_pH4.5_-CMC_C_, (c) GEL_pH8_-CMC_C_, (d) CA-CMC_C_, (e) CA-CMC_B_, and (f) CA-CMC_BT_ bilayer films. The arrows indicate the casein (purple) and carboxymethylcellulose (CMC) (red) layers.

### 3.3 Physicochemical Properties of Bilayer Films

The EMC and WVP values are shown in Table 1. The incorporation of carboxylic acids, such as citric acid and BTCA (Figure 1), into the polysaccharide matrix promotes the formation of intermolecular covalent bonds between polysaccharide hydroxyl groups and acid carboxyl groups through diester linkages (Figure S1). This cross-linking improves moisture resistance, which is particularly important given the high water solubility of CMC.^48^ The GEL-CMC films exhibited a reduced EMC of around 5% compared to CA-CMC films without TA (CA-CMC_C and B_), indicating limited availability of free polar groups to interact with water,^49^ and pH had no observable effect. Regarding WVP, the presence of charges appeared to hinder water permeation, especially in GEL_pH3_-CMC_C_, which showed a WVP 30% lower than that of GEL_pH4.5_-CMC_C_ and GEL_pH8_-CMC_C_. At high humidity, hydrogen bonds break, allowing water to intercalate between chains and layers, thereby enhancing the influence of electrostatic forces and resulting in stronger interactions with the CMC layer.^50^ CA-CMC_B_ demonstrated 20% lower WVP than CA-CMC_C_, indicating that BTCA is a more effective CMC cross-linker than citric acid. However, the high residual hydroxyl content in BTCA increases moisture retention, as reflected by a 25% rise in EMC. The CA-CMC_BT_ showed notable reductions in both WVP and EMC, supporting the idea of interfacial cross-linking. Interlayer cross-linking strengthens polymer interactions, thereby restricting moisture uptake at the interface and decreasing the number of polar groups available to interact with water.^51,52^ Regarding water affinity, biobased films generally exhibit higher water vapor permeability and moisture uptake than conventional commercial packaging materials (e.g., LDPE and PP),^53,54^ which is mainly attributed to their inherent chemical structure, particularly the presence of hydrophilic functional groups such as hydroxyl, carboxyl, and amino groups.^46^

**Table 1:** Equilibrium Moisture Content (EMC) and Water Vapor Permeability (WVP) Values of the Bilayer Films, and Water Contact Angle of Protein and CMC External Faces of the Bilayer Films^a^

			water contact angle (°)	
bilayers	EMC (%)	WVP (10^–6^·g·m·h^–1^·Pa^–1^)	protein face	CMC face
GEL_3_-CMC_C_	5.3 ± 0.8^a^	2.72 ± 0.04^a^	75 ± 4^af^	35 ± 3^b^
GEL_4.5_-CMC_C_	4.4 ± 0.2^a^	3.34 ± 0.08^b^	78 ± 1^ac^	36 ± 2^b^
GEL_8_-CMC_C_	6.6 ± 0.6^a^	3.1 ± 0.1^c^	92 ± 2^d^	46 ± 2^e^
CA-CMC_C_	30.7 ± 2.1^b^	3.71 ± 0.03^d^	74.4 ± 0.1^a^	43 ± 1^ac^
CA-CMC_B_	38.6 ± 0.7^c^	3.22 ± 0.04^e^	50 ± 5^e^	81 ± 3^c^
CA-CMC_BT_	8.3 ± 0.5^d^	2.1 ± 0.3^f^	68 ± 5^fg^	66 ± 4^g^

a Different letters indicate significant differences (*p* < 0.05) within the same analysis.

The water contact angle measurements (Table 1) showed different values for each polysaccharide and protein surface, indicating anisotropy in surface energy. Higher contact angle values indicate lower wettability and reduced interaction with water. The gelatin and casein faces exhibited comparable angles above 60°.^55^ Cross-linking CMC with BTCA resulted in a lower wettability of the polysaccharide layer compared to citric acid, as demonstrated by an increase in the contact angle from approximately 40° to 80°. This behavior is justified by the greater efficiency of BTCA in cross-linking CMC, making the hydrophilic groups capable of interacting with water unavailable.^56^ The addition of TA decreased the contact angle to 66°, as the polyphenol increased the hydrophilicity of the material due to its hydroxyl groups.^57^

The wettability of the films does not follow the same trend as EMC and WVP, suggesting that moisture absorption and permeability are not solely governed by surface phenomena, but may also be influenced by the bulk and interfacial characteristics. Considering the contact angle results for the CA-CMC systems containing BTCA, the CMC_BT_ face exhibited lower values than the CMC_B_ face, while the casein faces showed comparable wettability since the same material was used in these bilayers. Therefore, CA-CMC_BT_ system would be expected to show a greater interaction with water at the surface level. However, the EMC and WVP measurements revealed that the CA-CMC_BT_ bilayer exhibited the lowest moisture uptake compared to the CA-CMC_B_ and CA–CMC_C_. This behavior indicates the contribution of additional mechanisms beyond surface adsorption and secondary interactions, such as interfacial cross-linking, which restricts the free volume available for water molecules and remains effective even under high-humidity conditions.^46^

Water contact angle (θ) of the bilayer films depends on the outer biopolymer layer. Compared to polypropylene (PP, θ ∼ 104°)^58^ and low-density polyethylene (LDPE, θ ∼ 94°),^59^ the bilayer films are more hydrophilic, but their wettability is comparable to that of biodegradable polymers such as cellulose acetate (θ ∼ 70°)^60^ and polylactic acid (PLA, θ ∼ 60°),^61^ supporting their suitability for sustainable packaging applications.

The mechanical properties (Table 2) were determined by uniaxial tensile tests. The CA-CMC bilayer films exhibited layer delamination during the mechanical tests (Figure S2), while the other films remained intact. GEL_pH3_-CMC_C_ and GEL_pH8_-CMC_C_ showed a tensile strength of 20 MPa (Table 2), while GEL_pH4.5_-CMC_C_ presented a smaller value of 12 MPa, suggesting that the absence of net charges influences the mechanical performance of the system. The elongation at break of GEL-CMC bilayers, which reflects the material’s extensibility and relates to matrix cohesion (Table 2), was also reduced for the bilayer containing the gelatin at neutral pH (GEL_pH4.5_-CMC_C_), supporting this interpretation. As discussed by Goudie et al.,^62^ noncovalent interactions, such as electrostatic forces, influence the three-dimensional helical network structure of gelatin, which, in turn, relates to its mechanical properties. The mechanical performance of GEL-CMC films reflects the contributions of both layers, as the mechanical property values are within the ranges observed for the individual gelatin and CMC monolayers, and no delamination was observed during testing.

**Table 2:** Mechanical Properties, Tensile Strength (TS), Elongation at Break (EB), and Modulus of Elasticity (E), Weight Loss, and Onset Degradation Temperature (*T*_deg_) of the Bilayer Films^a^

bilayers	TS (MPa)	EB (%)	E (GPa)	weight loss (%)	*T*_deg_ (°C)
GEL_3_-CMC_C_	20 ± 4^ac^	5.0 ± 0.2^a^	0.6 ± 0.3^a^	10	251
GEL_4.5_-CMC_C_	12 ± 3^b^	2 ± 1^b^	0.6 ± 0.2^a^	7	249
GEL_8_-CMC_C_	20 ± 1^a^	4.7 ± 0.3^a^	0.9 ± 0.1^a^	9	258
CA-CMC_C_	23 ± 3^a^	4 ± 1^ab^	1.1 ± 0.2^a^	11	240
CA-CMC_B_	19 ± 2^a^	6 ± 1^a^	0.7 ± 0.1^a^	13	228
CA-CMC_BT_	15 ± 2^bc^	8 ± 1^c^	0.2 ± 0.2^a^	10	236

a Different letters in the same column indicate significant differences (*p* < 0.05).

Replacing citric acid with BTCA was expected to increase tensile strength and decrease elongation at break in CA-CMC films, due to the higher cross-linking efficiency of the CMC chains. However, an opposite trend was observed. According to the literature, this behavior can be attributed to a plasticizing effect caused by compounds rich in hydrophilic groups, such as BTCA, which promote moisture absorption.^63,64^ For CA-CMC_BT_, the reduction in tensile strength may be related to TA incorporation, which can cause a steric effect between CMC chains that weakens intermolecular interactions and increases chain mobility,^65,66^ as supported by the increased elongation at break. The CMC layer primarily determines the mechanical properties of bistructured films because the CA layer breaks early during testing. The tensile strength and elongation at break values of the CA and CMC monolayers (Table S2) are higher than the respective bilayers, suggesting that delamination caused defects that impair the mechanical performance of the films. The bilayer films containing gelatin and casein exhibited similar values of elastic modulus (E), indicating comparable stiffness under tensile stress.

The tensile strength and elastic modulus of the bilayer systems are within the range typically reported for polymeric films intended for flexible packaging applications. For instance, PP generally exhibits tensile strength values approximately 30 MPa, with elongation at break around 8.3%,^53^ while LDPE typically shows tensile strength values between 10 and 30 MPa and elongation at break ranging from 50 to 130%, depending on grade and processing conditions.^67,68^ These results indicate that the developed systems possess mechanically relevant properties for applications as a sustainable alternative.

The thermal behavior was evaluated by TG, as shown in Figure 6 and Table 2. Three main decomposition stages were observed: the initial weight loss between 25 and 130 °C is related to moisture desorption.^69^ The second stage, from 150 to 420 °C, is associated with the polymer decomposition through the breakage of peptide and glycosidic bonds.^27,70^ The decomposition temperatures of polysaccharides and proteins are known to differ, which together take place at a relatively wide temperature range (e.g., 200–430 °C for GEL-CMC films). The onset degradation temperature (*T*_deg_) was higher for the GEL_pH8_-CMC_C_ films, suggesting that electrostatic interactions and cohesion influence thermal stability. CA-CMC presented a lower *T*_deg_ than GEL-CMC systems, indicating that the protein layer makes a greater contribution to thermal stability. The CA-CMC_B_ and CA-CMC_BT_ films exhibited lower *T*_deg_ compared to the CA-CMC_C_, indicating that the presence of BTCA affected interchain interactions, as previously discussed in mechanical analysis.^22^ The third stage occurred between 440 and 600 °C and is associated with the decomposition of carbon-based functional groups, more pronounced in GEL-CMC systems.^27^

**Figure 6:**
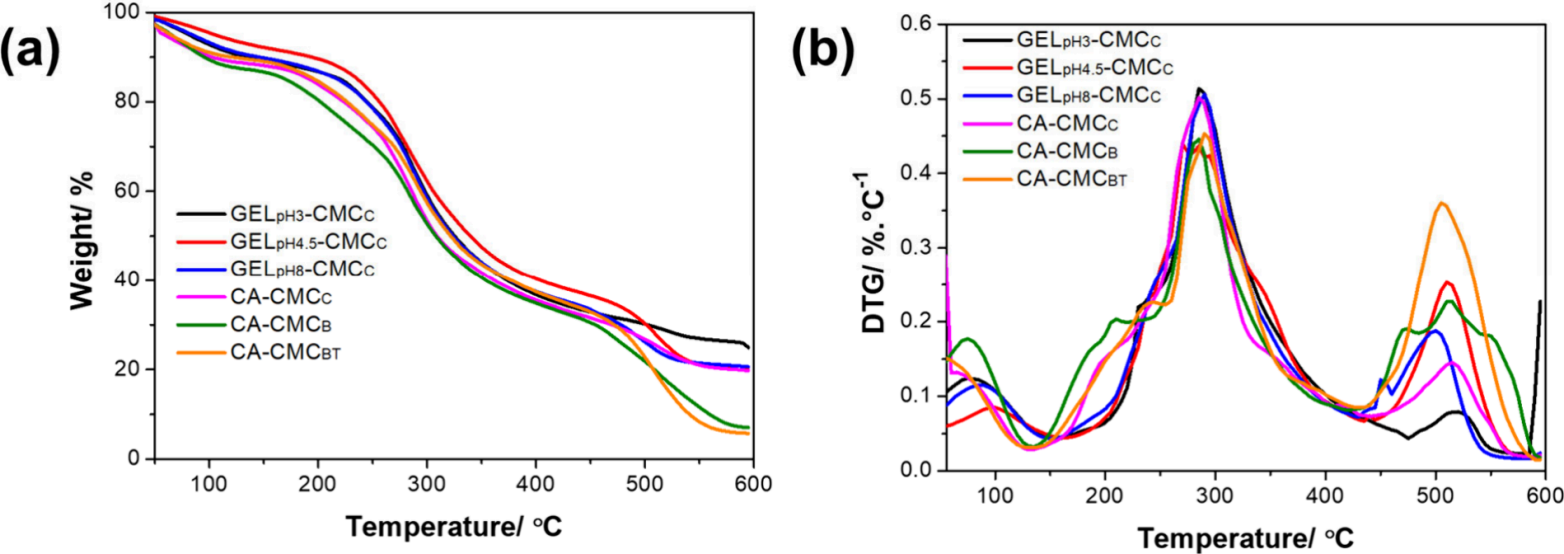
(a) Thermogravimetric (TG) and (b) derivative TG (DTG) profiles of the bilayer films.

The thermal degradation temperatures of the bilayer films are lower than those of conventional petroleum-based polymers, such as LDPE (*T*_deg_ ∼ 456 °C)^68^ and PP (*T*_deg_ ∼ 460 °C),^58^ as well as PLA (*T*_deg_ ∼ 380 °C).^71^ This behavior is typical of biopolymer-based systems due to the presence of thermally sensitive functional groups and lower molecular stability. Nevertheless, the degradation temperatures of the bilayer films remain well above typical processing and application temperatures for food packaging, indicating adequate thermal stability for their intended use.

## 4 Conclusions

Bilayer films composed of a gelatin or casein layer combined with a CMC layer were successfully produced by continuous casting, and their interfacial interactions were systematically investigated. QCM-D analysis demonstrated that CMC exhibited greater affinity for proteins with opposite (gelatin at pH 3) or neutral (gelatin at pH 4.5) charges than for negatively charged proteins (gelatin and casein at pH 8). The resulting bilayer films were self-supporting and optically transparent. GEL-CMC systems exhibited strong adhesion and could not be manually delaminated, whereas CA-CMC systems showed poor interlayer adhesion and were easily separated, regardless of whether BTCA or citric acid was used as the CMC cross-linker. The incorporation of TA with the BTCA (CA-CMC_BT_) promoted interfacial anchoring, yielding nondelaminable bilayers. Both GEL-CMC systems and CA-CMC_BT_ films exhibited low moisture affinity, as evidenced by their EMC values, attributed to enhanced interfacial adhesion that limits the diffusion and retention of water molecules between layers. Additionally, the films displayed anisotropic wettability, with the protein-layer faces being less hydrophilic than the CMC faces. The CMC cross-linker affected the properties of CA-CMC films, in which citric acid reduced water absorption, while BTCA improved water vapor barrier properties but decreased mechanical resistance. GEL-CMC had greater tensile strength than CA-CMC systems, and the pH of the gelatin layer did not significantly influence the mechanical performance. Overall, GEL-CMC bilayers demonstrated the greatest potential for food packaging applications compared to CA-CMC bilayers, owing to their strong interlayer interactions, lower water uptake, higher surface hydrophobicity of the protein layer, and improved thermal stability. Future work will focus on enhancing the water resistance through strategies such as surface modification and the incorporation of hydrophobic biobased additives to tailor surface energy and limit water–polymer interactions. These modifications are expected to broaden the applicability of the biobased multilayer films in the circular economy.

## Supplementary Material



## Notes

Values of frequency and energy dissipation from QCM-D analysis; Mechanical parameters of monolayer films; Image of uniaxial tensile test of bilayer film (PDF)
