# Supplementary material for: Protein–Polysaccharide Bilayer Films: Influence of Protein and Cross-Linker on Interfacial and Functional Properties
Source: Biomacromolecules. 2026 Feb 5;27(3):2103–13. doi: 10.1021/acs.biomac.5c02422 (PMC12977049; doi:10.1021/acs.biomac.5c02422)
Supplement: Supplementary file 1 [file bm5c02422_si_001.pdf]

Protein–Polysaccharide Bilayer Films: Influence of Protein and Crosslinker on Interfacial and Functional Properties

AUTHOR NAMES:

Giuliana T. Franco[a,b], *Luana Figueiredo*[a,c], *Caio G. Otoni*[d,e*], *Luiz H. C. Mattoso*[a,b,d*]

AUTHOR ADDRESS:

[a] Nanotechnology National Laboratory for Agriculture (LNNA), Embrapa Instrumentation – Rua XV de Novembro, 1452, São Carlos, SP 13561-206, Brazil;

[b] Graduate Program in Chemistry (PPGQ), Federal University of São Carlos (UFSCar) – Rod. Washington Luís, km 235, São Carlos, SP 13565-905, Brazil;

[c] São Carlos Institute of Chemistry (IQSC), University of São Paulo (USP) – Av. Trabalhador São Carlense, Parque Arnold Schimidt, São Carlos, SP 13566-590, Brazil.

[d] Graduate Program in Materials Science and Engineering (PPGCEM), Federal University of São Carlos – Rod. Washington Luís, km 235, São Carlos, SP 13565-905, Brazil;

[e] Institute of Chemistry, University of Campinas (Unicamp) – Rua Monteiro Lobato, 270, Campinas, SP 13083-862, Brazil.

CORRESPONDING AUTHORS:

Dr. Luiz H. C. Mattoso (E-mail: luiz.mattoso@embrapa.br; Phone: +55 16 21072827)

Dr. Caio G. Otoni (E-mail: cgotoni@unicamp.br; Phone: +55 19 35213054)

KEYWORDS: bilayer film; crosslinkers; interfacial interaction; interlayer adhesion; biopolymers combination.

Supporting Information

Table S1. *Values of the frequency ($\Delta f_{CMC\text{-}protein}$) and energy dissipation ($\Delta D_{CMC\text{-}protein}$) differences between the injections of the carboxymethylcellulose (CMC) and the protein solutions. The $\Delta D/(-\Delta f)$ corresponds to the ratio of the $\Delta D_{CMC\text{-}protein}$ and ($-\Delta f_{CMC\text{-}protein}$), which enables the evaluation of the rigidity of the deposited layer on the sensor.*

Systems	***$\Delta f_{CMC\text{-}protein}$/ Hz***	***$\Delta D_{CMC\text{-}protein}$/ 1.10^{-6}***	***$\Delta D/(-\Delta f)$/ $1.10^{-6}.Hz^{-1}$***
Gelatin pH 3/ CMC	-1	4	3
Gelatin pH 4.5/ CMC	-4	20	5
Gelatin pH 8/ CMC	-107	0	0.003
Casein/ CMC	4	-2	0.4

Table S2. *Tensile strength (TS), elongation at the break (EB), and modulus of elasticity (E) values of the monolayer films. Different letters in the same column indicate significant differences ($p<0.05$).*

**Monolayers**	**TS/ MPa**	**EB/ %**	**E/ GPa**
CMC-C	27 ± 7^{a}	3 ± 1^{a}	1.5 ± 0.1^{a}
CMC-B	25 ± 4^{a}	1 ± 1^{a}	1.6 ± 0.2^{a}
CMC-BT	20 ± 1^{a}	4.00 ± 0.02^{a}	0.95 ± 0.03^{b}
$GEL_{pH\ 3}$	14 ± 2^{b}	3.4 ± 0.1^{a}	1.0 ± 0.2^{b}
$GEL_{pH\ 4.5}$	20 ± 2^{a}	8 ± 1^{b}	0.9 ± 0.1^{b}
$GEL_{pH\ 8}$	25 ± 4^{a}	6 ± 1^{c}	1.2 ± 0.3^{ab}
CA	15 ± 1^{b}	6 ± 1^{c}	0.7 ± 0.1^{c}

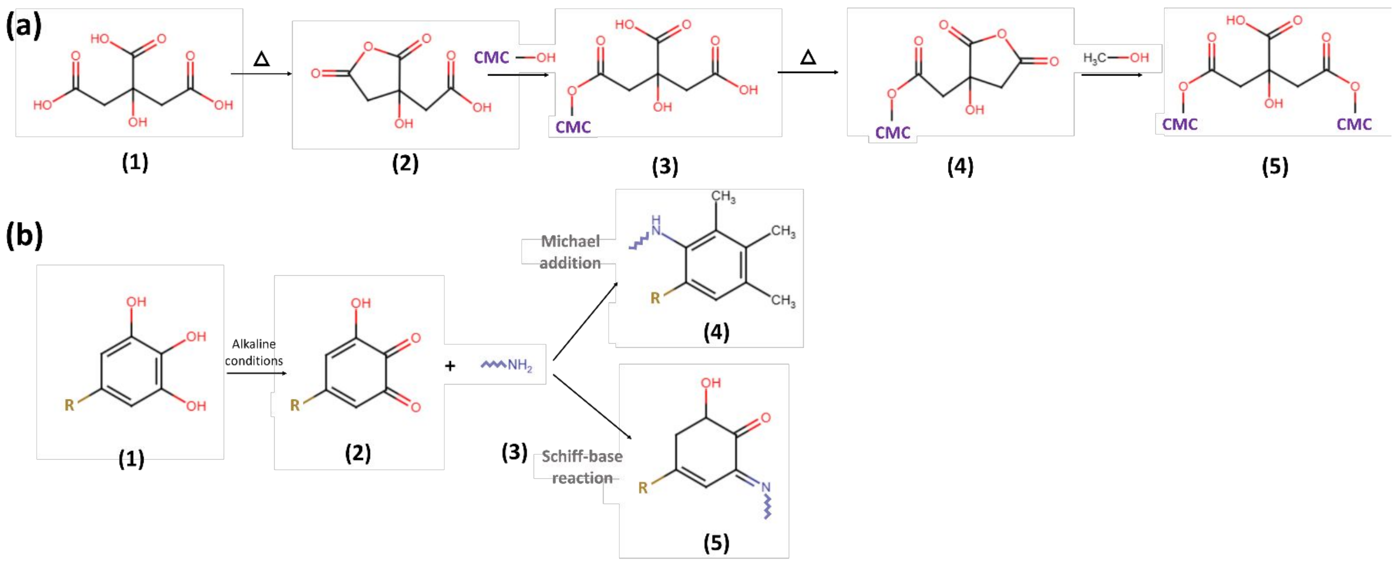

Figure S1. *(a) Schematic representation of the crosslinking reaction mechanism between carboxymethylcellulose (CMC) and citric acid (1). Upon heating, citric acid undergoes a condensation reaction to form a cyclic anhydride (2). This reactive intermediate then esterifies with the hydroxyl groups of CMC (3), forming an ester bond. Subsequently, a second condensation (4) followed by an esterification reaction occurs between the anhydride and another CMC chain (5), resulting in intermolecular crosslinking. (b) For the schematic representation of the crosslinking mechanism between tannic acid and a protein, tannic acid (1) is initially oxidized under alkaline conditions to form quinone intermediates (2). These intermediates can react with primary amine groups of proteins (3) via either Michael addition (4) or Schiff base (5) reactions, resulting in the formation of new C–N covalent bonds.*

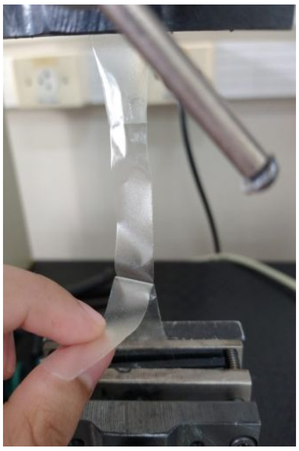

Figure S2. *Representative image of the delamination observed during the uniaxial tensile test of CA-CMC bilayer films.*
